# Climate and mental health: A roadmap to global heat resilience

**DOI:** 10.1371/journal.pmen.0000111

**Published:** 2024-08-15

**Authors:** Thomas E. J. Addison, Andrew Mackenzie, Alessandro Massazza, Lauren Mills, Shania Pande, Catherine L. Sebastian, Madeleine C. Thomson, Mike J. Tipton, Winnie Wefelmeyer

**Affiliations:** 1 The Physiological Society, London, United Kingdom; 2 Wellcome Trust, London, United Kingdom; 3 Extreme Environments Laboratory, University of Portsmouth, Portsmouth, United Kingdom; PLOS: Public Library of Science, UNITED KINGDOM OF GREAT BRITAIN AND NORTHERN IRELAND

## Climate change, rising heat and mental health

Climate change has been described by the World Health Organization as ‘the single biggest health threat facing humanity’ [1]. One consequence of human-driven climate change is that extreme heat events are no longer rare occurrences but are becoming more frequent, intense, and prolonged, posing a direct threat to human life and health. Global average temperatures are also increasing across the world.

These extreme ambient temperatures are associated with worsened mental health. According to the Intergovernmental Panel on Climate Change’s (IPCC) 2023 report, there is a ‘*very high confidence* that climate change has already negatively impacted mental health globally’ [2]. The impacts of heat on mental health include, but are not limited to, increased hospital attendance or admissions for mental health problems, increased suicide attempts and an increase in overall mortality [3, 4]. This is on top of existing contributors to the existing burden of mental health problems globally which are widespread, undertreated and under-resourced [5].

It is worth reiterating that without a rapid and just transition away from fossil fuels, our ability to adapt to the effects of heat on mental health will be overtaken.

## Mental health and the climate crisis

Mental health is an often unnoticed and under-researched aspect of the climate crisis. For example, a report by the Lancet Countdown Europe found that only 5% of the coverage around the health impacts of anthropogenic climate change in scientific articles in Europe was found to focus on mental health (versus 31% that focused on infectious diseases and 18% on cardiorespiratory diseases) [6]. While the association between heat and mental health problems is beginning to be recognised by researchers and policymakers, the mechanistic links between the two remain poorly understood and under-researched. Suggested reasons include: the challenge of untangling the multiple causes and drivers of poorer mental health during and following heat events [7], the variety of stakeholders involved in developing and implementing solutions [8], a lack of reliable data from the continents that most regularly experience high temperatures such as Africa [9] and stigma and minimisation limiting our response to mental health problems more broadly [10].

Two major shifts are occurring in response to this historical lack of focus on mental health and the climate crisis. Firstly, there is increasing recognition of, and interest in, the growing challenge presented by mental health problems in a rapidly warming world and how this growing challenge intersects and catalyses the existing burden on mental health globally [11]. Secondly, while physical health is now much more prominent as part of the climate change debate, there is insufficient research or policy overlap between mental and physical health, and even less that seeks to gain a better understanding of the physiological mechanisms that link rising temperatures to mental health problems.

## Response from our organisations to date

In addition to Wellcome’s and The Physiological Society’s work on the impact of heat on physical health, we are moving quickly to prioritise the relationship between heat and mental health. As Wellcome notes, ‘Climate change is causing temperatures around the world to rise, which can have a variety of detrimental impacts on mental health’ [12]. The Physiological Society’s 2023 *Heat Resilience Strategy* report included a call for the UK Government and devolved administrations to urgently develop a *National Heat Resilience Strategy* that is rooted in physiology to protect vulnerable populations from the health impacts of extreme heat including those living with mental health problems.

While the correlation between heat and mental health problems is clear, the physiological mechanisms driving these worsened outcomes are under-researched and poorly understood. However, various potential biological, psychological and social mechanisms have been proposed and require further exploration and research. These include but are not limited to the impact of heat on mental health via worsened sleep and mood, disrupted routines and coping mechanisms such as spending time outdoors, or due to the potential impact of some psychotropic medications on body thermoregulation. Physiological interventions are an essential part of the solutions that will need to be developed to address these mechanisms. For example, understanding how heat affects our sleep and the long-term implications of the body’s response, would improve not only our physiological interventions but also address some of the psychological and social drivers of poorer outcomes. Many within the physiological community feel that too often physiology has been missing from both mental health and climate change discussions with inevitable negative consequences in terms of possible solutions.

## Responding to unmet needs and identifying research gaps

In response to the unmet needs that have been identified, The Physiological Society and Wellcome held a joint workshop in February 2024, *Bringing mechanistic understanding and real-world impact to the link between extreme heat and mental health*. This workshop brought together 80 experts from over 16 countries around the world and a variety of academic disciplines such as physiologists, epidemiologists, climate change experts, mental health practitioners, people with lived experience and pharmacologists. The workshop covered topics from current research efforts to understanding the biological, psychological and social interactions between poorer mental health and heat, to exploring government policies that could help to improve the speed of implementation of research insights.

## Key findings and recommendations from the workshop

### There is a need for better understanding of the physiological mechanisms underpinning the association between extreme heat and mental health

There is an urgent need to address research gaps in the understanding of the physiological mechanisms linking extreme heat to poorer mental health outcomes. This includes exploring physiological changes, neurotransmitter involvement, and neuro-developmental impacts. This will give us a greater understanding of these conditions and ultimately help to design effective interventions and adaptations.

### Borrow designs from the field of physiology to investigate the mechanisms underpinning the association between heat and mental health problems

An improved overall understanding of the mechanistic pathways linking heat to poorer mental health outcomes would enable prioritisation of research and thereby better use of the finite resources available for such research.

## A greater focus on fostering transdisciplinary and collaborative research

### Promote collaboration in research design through consortia-based funding models

Funding bodies should focus on achieving a balance between mechanistic research and larger-scale intervention studies. Funding bodies can encourage collaboration among researchers by funding projects that require multidisciplinary or interdisciplinary approaches, including physiologists.

### Improve the use and integration of existing mental health and weather and climate data

Existing mental health records and weather and climate databases should be used to study the impact of different heat indices on mental health; these data could inform physiological adaptations to heat and related mental health problems. However, significant barriers exist such as the lack of availability of mental health data globally and the limited spatial and temporal resolution of mental health data, when compared with weather or climate data.

## Equitable access

### Ensure a concerted focus on mental health problems associated with extreme heat low-and middle-income countries (LMICs)

Research consortia often neglect to include regions where most extreme heat events occur or that are chronically exposed to high temperatures and where communities are most vulnerable to its impacts, such as in low and middle-income countries closest to the equator. This neglect may lead to both disparities in understanding and addressing mental health problems in these contexts.

### Implement simple, cheap and sustainable cooling interventions and evaluate their impacts on mental health

Implementing simple, cheap and low-carbon interventions like using fans to keep people cool should be promoted widely, although currently they are not widely included in government guidance and there is a lack of evidence for their impact on mental health. This should be implemented as part of a trial, or accompanied by research monitoring the effects of these interventions, as evidence for their impact on mental health is currently lacking.

## Our future work

As a next step, we are working on ways to build communities and opportunities in this space. Wellcome is working on a funding call to understand the mechanistic links between heat and worsening mental health, at the physiological, psychological and social level.

Meanwhile, The Physiological Society has been working with other organisations from both mental health and climate-focused backgrounds, to develop a roadmap for change to help inform the thinking of politicians and policymakers throughout the world. This roadmap was officially launched as part of an event in the Houses of Parliament. The event was designed to discuss how we can best bring together academics who provide the evidence, with politicians who will decide how effective policies are developed and implemented to protect us, and the planet, against the worst effects of climate change.

Just as an effective response to the climate emergency requires action at all levels and throughout the world, developing an effective approach to understanding and addressing the link between heat and mental health problems will require new collaborations and solutions to emerge. Our organisations stand ready to support these endeavours.
